# Case of Abscess of the Female Breast, from Which a Worm Was Discharged

**Published:** 1847-02

**Authors:** 


### ARTICLE IV.

### *Case of Abscess of the Female Breast, from which a Worm was discharged.*

Dr. Thompson Mead, of Kane Co., Ills., has related to us this case, which, as far as we know, or can learn from the examination of any works within our reach, is unique in the records of medicine.

A young woman, in good health, of about 20 years of age, was, during the past autumn, while nursing her first child, attacked with pain and induration of the breast, which, after a few days, resulted in an abscess, which was punctured. Pus, mixed with blood, was discharged in considerable quantity, after which the Doctor perceived a small body protruding, and, seizing it with his forceps, drew it out. It proved to be a worm nearly an inch in length, of a yellow color, jointed, pointed at the extremities, firm in texture, and extremely active and tenacious of life. The abscess soon healed, but, at the time we saw the Doctor, another seemed to be forming at a different point of the breast.

In what way could the worm have found its way to that situation? The only possible solution of that question which occurred to us was, that it might have been generated in the intestinal canal of the child, and passed from its mouth to the breast while the infant was asleep, or holding the nipple in its mouth without sucking. This is rendered somewhat probable by the fact, that worms have been found in the nasal cavities, antrum, frontal sinuses, &c., derived from that source. The child was nearly a year old at the time of the formation of the abscess, which further adds to the probability of the conjecture. If there is some other and better explanation to be found, we will feel obliged to any one who will furnish it to us.

The worm, corresponding to the description given, has been deposited by Dr. Mead in the museum of Rush Medical College. D. B.

*Chicago, Dec.* 21, 1846.

